# Left atrial giant thrombus infected by Escherichia Coli. Case report

**DOI:** 10.1186/1749-8090-3-18

**Published:** 2008-04-23

**Authors:** Panagiotis Dedeilias, Antonios Roussakis, Efstratios N Koletsis, Dimitrios Zervakis, Panagiotis Hountis, Christos Prokakis, Christina Balaka, Konstantinos Bolos

**Affiliations:** 1 Cardiac Surgery Department, Evangelismos General Hospital, Athens, Greece; 2 Cardiothoracic Surgery Department, University of Patras, Greece

## Abstract

**Background:**

Left atrial thrombi are mostly related to mitral valve disease. The differential diagnosis of clots and myxomas in the left atrium is mostly based on echocardiography. Infection of intracardiac thrombi is extremely rare and mostly reported in ventricular clots or aneurysms following myocardial infarction.

**Case presentation:**

We present the case of a 65 year old female with a history of mitral valve disease and chronic atrial fibrillation who suffered repeated embolic strokes and a giant infected clot in the left atrium. Although the patient underwent prompt surgery with removal of the clot and valve replacement the complication of septic emboli to the CNS led her to death. To the best of our knowledge this is the second report of an infected left atrial thrombus.

**Conclusion:**

The case is a representative example of a neglected and undertreated patient with catastrophic consequences. Anticoagulant therapy in patients with mitral valve disease and atrial fibrillation should be applied according the currently available guidelines and standards in order to avoid analogous paradigms in the future. Mitral valve substitution should be considered in patients with mitral valve disease presenting thromboembolic complications. Surgery should be considered as the treatment of choice in cases of organized left atrial thrombus and suspected tumor or infected mass.

## Background

Cardiac mural thrombosis is a complication of mitral valve disease frequently related to systemic thromboembolism. Infected intracardiac thrombi are extremely rare and mostly reported in ventricular chamber. So far there has been only one case of an infected thrombus in the left atrium [1]. This report describes the case of a patient with a history of mitral valve disease and atrial fibrillation with repeated embolic strokes in the past presenting a giant infected clot in the left atrium. Although the patient underwent surgery and removal of the clot the frequency and the severity of the strokes along with the complications of septic emboli to the CNS led her to death.

## Case presentation

**Figure 1:**
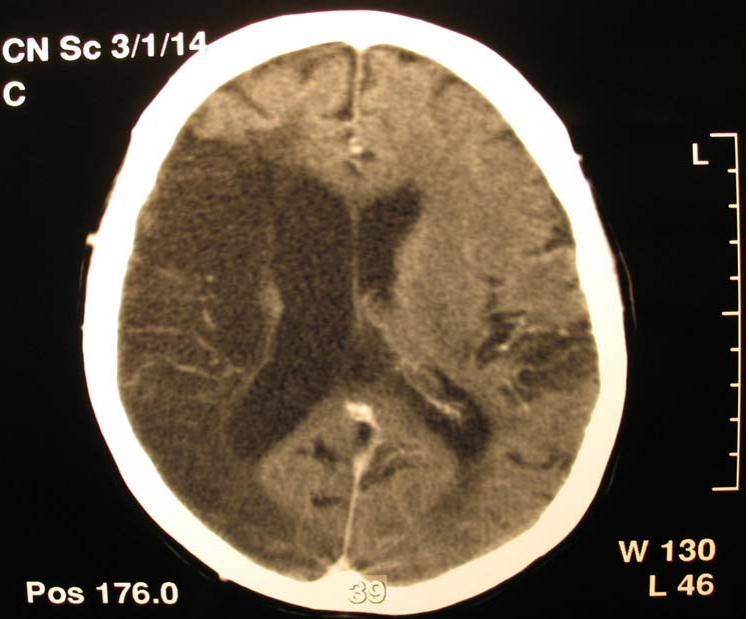
**Preoperative CT brain scan Sample figure title**. CT brain scan-Old ischemic findings in the right hemisphere.

**Figure 2:**
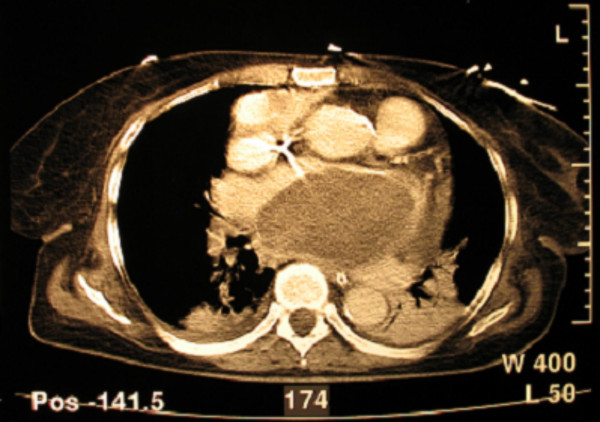
**Preoperative C-T Thoracic Scan**. C-T Thoracic Scan – Large mass in the left atrium without enhancement.

**Figure 3:**
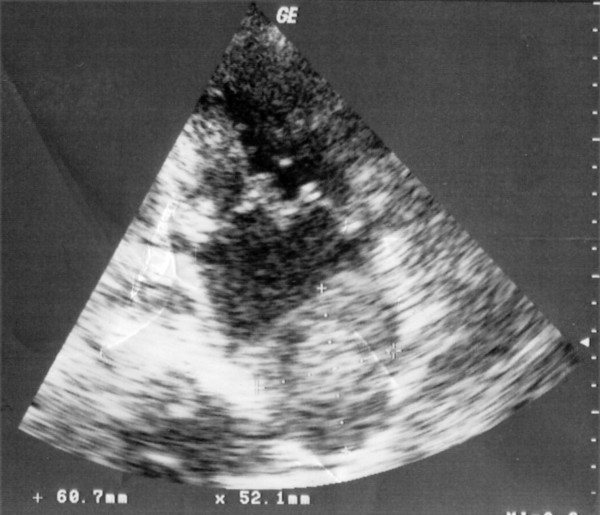
**Preoperative echocardiography**. Echocardiographic confirmation of the large mass in the ventricle of left atrium.

A 65 year old woman was admitted to our hospital with disturbances of consciousness ensued during the last two days. She had been suffering from mitral stenosis for 15 years and was under medical supervision, receiving digoxin, furosemide and warfarin. She had suffered three embolic strokes over the last 5 years. At present the first clinical examination revealed Glasgow Coma Scale (GCS) of 6, left hemiplegia, atrial fibrillation, low blood pressure of 70/40 mmHg and temperature of 38.5°C. With the diagnosis of an imminent stroke the patient underwent emergency CT scan of the brain and thorax. Brain scan disclosed extensive ischemic damage to the right brain hemisphere (Figure 1), compatible with preexisting lesions. The CT of the thorax revealed an enlarged left atrium and the existence of a distinctly outlined mass of 10.3 cm × 6.3 cm × 6.5 cm in the left atrium (Figure 2) Echocardiography confirmed the presence of the mass and severe mitral valve stenosis with valve opening of <1 cm^2 ^(Figure 3)

**Figure 4:**
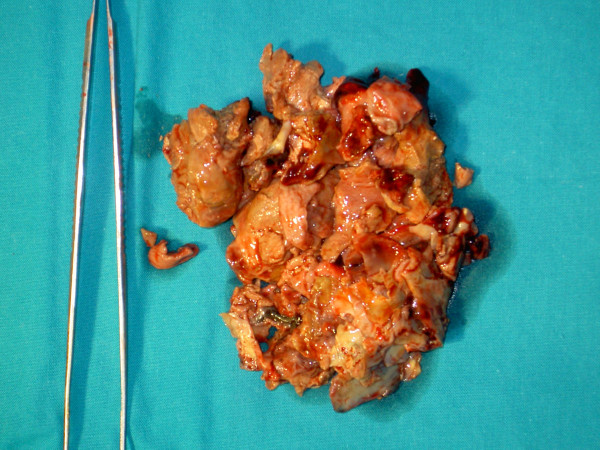
**Infected thrombus specimen**. The extracted brittle and foul-smelling mass.

The patient was urgently referred to our cardiac surgery department with the differential diagnosis of a giant left atrial thrombus or left atrial myxoma and underwent removal of the mass through a transatrial approach. The mass was locally semi-liquid and foul-smelling, giving the impression of a huge infected thrombus (Figure 4). The mitral valve was severely calcified and was replaced by a metallic prosthetic valve.

**Figure 5:**
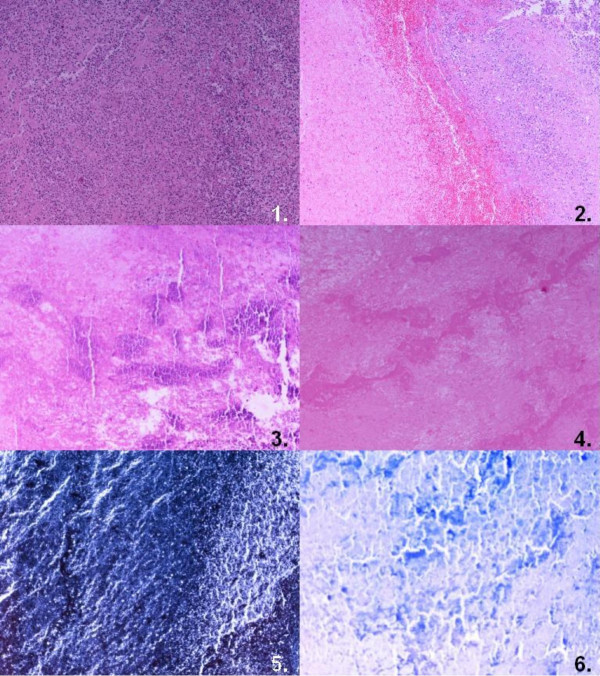
**Histology specimens of the thrombus**. 1. Inflammatory material from left atrium ventricle (H-E ×40), 2. Thrombotic material from the same area (H-E ×40), 3. Deposits of calcium salts (H-E ×40), 4. Necrotic alterations (H-E ×40), 5. Absence of microorganisms (Histochemical Grocott-Gomori stain) (×40), 6. Absence of microorganisms (Histochemical Giemsa stain) (×40).

**Figure 6:**
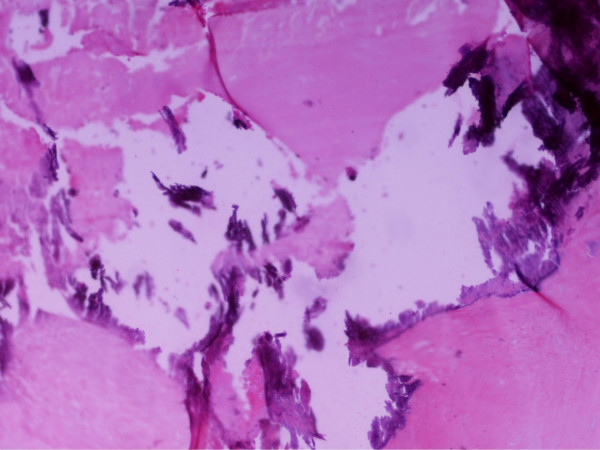
**Mitral valve histology specimen**. Mitral valve with degenerative alterations (H-E ×40).

Histological examination confirmed the diagnosis, revealing clot material with numerous polymorphoneuclear cells (Figure 5). The extracted mitral valve also revealed minor inflammatory infiltration (Figure 6). Blood and specimen cultures grew Escherichia Coli, with multisensitivity profile, compatible with community-acquired infection.

She was admitted to the ICU in a state of shock and anuria, receiving high doses of inotropic drugs and antibiotic treatment with ceftriaxone, gentamycin and metronidazole. She was also put under full anticoagulant therapy. A new transoesophageal echocardiogram in the 7^th ^post-operative day disclosed recurrence of thrombus remnants in the left atrial appendage. After 10 days she was weaned of the inotropic drugs and the veno-venous hemofiltration, and 15 days later she was also weaned of the ventilator. The patient during this period was afebrile. After 3 days of spontaneous breathing she had to be re-intubated because of progressive respiratory insufficiency due to intervening further neurological deterioration and fever reappearance. Lumbar puncture revealed findings consistent with a central nervous system infection, probably due to septic emboli. Antibiotic treatment was then switched to meropenem, but it was unsuccessful. The patient died 5 days later due to brain stem herniation.

## Discussion

Left atrial thrombi associated with mitral valve disease represent significant risk factor for systemic thromboembolism and sudden death [2]. Such sizable thrombi as the one we present have sometimes been mentioned in case reports [3-5] but only one case described by Fukuchi et al [5] refers to a patient receiving continuous anticoagulant therapy.

Diagnosis is often made with chest computed tomography as in our case although transoesophageal or 3-d transthoracic echocardiogram are known to be superior in identifying left atrium masses and clarifying the diagnosis between thrombus and left atrial myxoma [6-8]. Cardiac MRI is comparable to TEE in the detection of atrial thrombi, especially in the left atrial appendage, but it is time consuming and carries a high cost precluding its use as a routine procedure [9].

Definite diagnosis requires histological confirmation, as there have been some case reports describing development of atrial myxoma in the setting of an already existing mitral stenosis [7,10]. Therefore surgery should always be considered in these patients if the differential diagnosis between myxomas and clot in the left atrium is not possible despite the use of echocardiogram and CT scan. Another indication for surgical treatment is the infection of the clot as in our patient. Conforming to our management Okayama et al reported surgical management of an infected left atrial thrombus in a male patient who wasn't under anticoagulant therapy [1]. The kind of microorganism causing the infection is commented on by the authors in this last case as being similar to other reports, referring mainly to infected ventricular thrombi. As there are no reports about gram-positive intracardiac thrombus infections, it is probable that the natural history and related pathogens differ from native or prosthetic valve infections. Surgery should also be undertaken if an organized thrombus is identified at the echo examination since conservative management with anticoagulants may lead to an unpredictable pattern of thrombus resolution and formation of fragments that may result in systemic emboli [11-13].

Medical therapy consisting in anticoagulants has been proposed for sessile, immobile thrombi presenting less risk for thromboemblic complications in candidates for percutaneous transvenous mitral commissurotomy [14]. Thrombolytic therapy as well as anticoagulant could be used only for unorganized thrombi [15]. So far there have been few reports of thrombolytic therapy for left atrial thrombi in the literature [15,16].

Large atrial thrombi are very rare as the incidence of native valve disease tends to be reduced and the anticoagulant therapy is evolving and becoming more standardized [17]. Additional infection of a thrombus is even rarer, as guidelines about the use of prophylactic antibiotics in this group of patients are now generally applied.

## Conclusion

The case report presented is a representative example of a neglected and undertreated patient in refers to both surgical and medical aspects of her disease with extreme complications. In order to avoid such references in the future anticoagulant therapy must be used according to the currently recommended guidelines and standards and surgery must be considered in the setting of mitral valve disease complicated by systemic thromboemolism. In the presence of left atrial thrombus surgery should be offered in all patients with organized thrombus or suspicion of a tumor or an infected mass.

## List of abbreviations

CNS: Central Nervous System. GCS: Glasgow Coma Scale.

## Competing interests

The authors declare that they have no competing interests.

## Authors' contributions

All authors: 1) have made substantial contributions to conception and design, or acquisition of data, or analysis and interpretation of data; 2) have been involved in drafting the manuscript or revising it critically for important intellectual content; and 3) have given final approval of the version to be published
